# What We “Cannot *Not* Know in America”: 400 Years of Inequality and Seven Sins

**DOI:** 10.3389/fpubh.2021.678053

**Published:** 2021-06-15

**Authors:** Robert Sember, Mindy Thompson Fullilove, Robert E. Fullilove

**Affiliations:** ^1^The New School, New York, NY, United States; ^2^Mailman School of Public Health, Columbia University Irving Medical Center, New York, NY, United States

**Keywords:** racism, inequality, 1619, anniversary, police violence, COVID-19, neoliberalism

## Abstract

The 400 Years of Inequality Project was created to call organizations to observe the 400th anniversary of the first Africans landing in Jamestown in 1619. The project focused on the broad ramifications of inequality. Used as a justification of chattel slavery, structures of inequality continue to condition the lives of many groups in the US. Over 110 organizations joined this observance and held 150 events. The highlight of the year was the homily given by Reverend William Barber II, co-chair of the Poor People's Campaign, who described the “seven sins” that link the concept of inequality to every aspect of national life, from politics to militia. These “seven sins” help us to analyze and address crises, such as the COVID pandemic.

The year 2020 will be remembered for two epoch-defining events: a pandemic on a scale not seen for more than 100 years, and the largest uprisings for racial justice in US history. The demonstrations against police violence organized by Black Lives Matter and other movement coalitions in cities across the U.S. were rapidly joined by an unprecedented number of sympathetic demonstrations in countries across the world. Both events underscored how systemic and frequently race-based inequalities condition local, national, regional, and global health-related vulnerabilities. As some have noted, there is an apocalyptic quality to this moment: a veil is being pulled back to reveal, what Cornel West has termed, “a reality that one cannot *not* know” (1). Camara Phyllis Jones, former President of the American Public Health Association, finds this “both a treacherous time and a time of great promise” (2). The stark racial disparities in COVID-related deaths—Black Americans have been dying at more than twice the rate of white people (3)—affirm again the deadly consequences of racism, a fact Jones underlines with her declaration that, “Race doesn't put you at higher risk. *Racism* puts you at high risk” (emphasis in the original). Heartened that “folks from all parts of our population,” expressed outrage following the murder of George Floyd, Jones wonders, cautiously, if, “perhaps this nation is awakening to the realization that racism does indeed hurt us all.” If “perhaps” this is a time of awakening, might it now be possible to change paths and head in the direction of the solution, if only we knew what that was?

For over 30 years we have examined the determinants of a number of health concerns in the US. Our investigations of the excess risk for AIDS among Blacks and Hispanics, for example, revealed how urban renewal and other forms of neoliberal disinvestment fractured communities and thinned the supports necessary to sustain life, community, and spirit (4–6). In demonstrating the constitutive role geographies of inequality have on community and population health, our research drew from an added work of medical and health geographers such as Pyle (7), Dear and Wolch (8), and Brown et al. (9). This body of work ilustrates how, on the one hand, the diffusion of disease is facilited by racial and other forms of segregation, which, on the other hand, curtial the distribution of and access to care. We saw this process repeated as poor and minority communities faced the epidemics of crack cocaine addiction, violence and associated mental disorders, multi-drug resistant tuberculosis, and asthma (10–12). Now, these geographies of inequality and other social fractures are shaping the COVID-19 pandemic. Thus, while this moment is extraordinary, it is not unprecedented. Indeed, some of its deepest lessons lie in what is familiar.

As with past epidemics, the burdens of COVID—infections, deaths, access to care, vaccination rates, and the host of associated ills such as housing and food insecurity and occupational hazards—fall inequitably on poor, low wealth, and majority communities of color (13, 14). Similarly, the murders of George Floyd, Breonna Taylor, Ahmaud Arbery, and so many others by police officers and white supremacist vigilantes are grim additions to a legacy that is centuries deep. We also face a familiar contradiction: the corporate and state systems that have failed us, that have, in fact, helped produce and amplify these crises, are also the very ones we most need in this moment because of their scale, reach, and resources (15).

Knowing what we cannot *not* know, our task in this apocalyptic moment is 2-fold: address the immediate and urgent issues of life, death, and justice, while also transforming our institutions so that we are better able to meet the challenges of the future. This is a complex undertaking for the goal is not to “return to normal.” The history of the AIDS crisis offers a cautionary tale in this regard. In a 1993, a special panel convened by the National Academy of Sciences, Engineering, and Medicine to monitor the social impact of the AIDS crisis, concluded that, “Although the AIDS epidemic has devastated the lives of many individuals over the last decade, there is little evidence that six major American social institutions [the health care delivery system, the public health system, correctional systems, voluntary and community-based organizations, and religious groups] have been changed fundamentally” (16). Rather, they concluded, AIDS had become, “accepted as one of the ‘synergy of plagues' endemic in vulnerable communities.”

Inherent in that conclusion was the assumption that endemic disease was not only concentrated but also contained in poor and minority communities. That assumption was incorrect: disease, dysfunction and social pathology have spread broadly across the nation from the places where they were allowed to fester. The epidemic of “deaths of despair” among white working-class people in the Rust Belt, for example, is part of the insidious march of disease across geographic space and up the social hierarchy (17). This process has been orchestrated over the past three decades by neoliberal “reforms” that devolve the responsibilities of the state to “the market,” eliminating, in the process, programs that provided some relief to poor and low wealth families and communities. The US's bifurcated health care system, a patchwork of profit-driven and public funded enties that leaves millions without affordable or secure access to healthcare, is perpetuated by these policies. First adopted by the Chilean dictator General Augusto Pinochet and then by Prime Minister Margaret Thatcher in Great Britain and President Ronald Reagan in the US, neoliberalism is now a global economic and governance system. The resulting policies and practices are an underlying driver of the COVID pandemic where poor and other marginalized populations are abandoned and ignored (18). Can we learn from this disaster and move in a different direction? To answer this question, we reflect on what we learned from Reverend William Barber II's homily on the seven sins of the United States.

## 400 Years of Inequality

In 2016 we launched the 400 Years of Inequality Project, a call for observance of the 1619 landing of Africans at Jamestown to be sold into bondage. Anniversaries are widely recognized as events with individual and collective meaning (19). Their observance is a part of ritual and culture (20). In addition to observing the anniversary and reflecting on the past, we called for a “People's Platform for Equity.” If the observances provided opportunities for communities to name the problem, designing the People's Platform for Equity would be the “intervention.” Together, these activities would affirm what we stand for and where we are headed. To that end we planned from the beginning of the project a Solemn Occasion that would assimilate the insights of 400 years of inequality into a blueprint for the future. Our dream was that Rev. William Barber, co-chair with Rev. Liz Theoharis of the Poor People's Campaign, would deliver this message from the pulpit of Riverside Church in New York City where prominent national and internation leaders have provided guidance in other times of crisis.

We chose to focus our work on “inequality,” arguing that the slaveholders invented tools of dehumanization that they used to justify slavery and that have been used ever since to oppress people along lines of race, class, gender, religion, immigration status, sexual orientation, and many other dimensions. As we worked together to develop tools for the anniversary observances, we came to think of the system that arose from those dehumanizing foundations as an, “ecology of inequality.” Massey and Eggers, in a groundbreaking paper on the topic of the ecology of inequality, looked at the ways in which public policy had created spatial isolation of poor Blacks and Hispanics (21). Other policies disrupted the economic basis of American life through deindustrialization, the transformation of farming to agribusiness and the divergence of employment in high-paying tech sectors and low-paying service sectors. This understanding of an ecology—a life and social system in which we are all engaged, both those with the greatest privilege and those with the least—was a shift in our thinking, and guided the way in which we approach the creation of a People's Platform.

The 400 Years of Inequality Project was launched by five partner organizations: the University of Orange, The New School, ONE DC, Columbia University Mailman School of Public Health, and Voices of a People's History. Our first task was to increase awareness of the anniversary. In 2017, we established a website and developed the project logo, the number 400 with a loop of chain linking each digit (22). The organizing committee suggested that individuals or local groups research and observe local histories of and movements against inequality. This place-based focus would enable observance participants to develop conversations regarding the history of inequality they, their neighbors, and ancestors had experienced and witnessed.

We asked observance organizers to let us know about events. We heard from 110 organizations across the country. Since a number of them mounted multiple observance events, we received information on close to 150 events. We were also aware of multiple organizations organizing events that did not submit information to us. While we are not able to provide a full accounting of observances, we are confident that they number in the hundreds.

Participating organizations ranged from CBO/NGOs and religious groups to academic institutions, schools, and museums and historical societies. Black civil rights organizations and black churches were expected to participate in large numbers and they did. What was more surprising was the extent to which “mainstream” organizations of major stature participated. These included: Carnegie Hall, the Brooklyn Public Library, the American Journal of Public Health, and schools of public health. We documented events at Tulane University's School of Public Health and Tropical Medicine, the Mailman School of Public Health in New York City, the School of Public Health at Boston University, Harvard University's T.H. Chan School of Public Health, and The Drexel University Dornsife School of Public Health. Dr. Sharrelle Barber, Rev. William Barber II's daughter, organized the event at Drexel University.

Observance themes ranged from the legacies of slavery, to the history of urban segregation, and present-day health inequities. They were addressed in conferences, symposia, worship services, exhibitions, and community celebrations. These events wove together multiple strands of history to illustrate how different communities and events have been caught up in the processes of inequality and have organized against them. Residents of a neighborhood in New York City organized a candlelight vigil at the site of a recently discovered African burial ground. African immigrants and Native American residents from the neighborhood joined local faith leaders and community members for prayers and reflection. The event inspired the organizers to form a neighborhood racial justice committee and the community is involved in planning a memorial for the site. In “The Cotton Series,” dancer and choreographer Havanna Fisher and her ensemble drew from the history of cultivating and harvesting cotton and sewing with cotton fabrics to explore the living and working worlds of black women, beginning with African ancestral practices to present-day celebrations and justice struggles. The Allen Memorial Art Museum at Oberlin College mounted the exhibition, “Afterlives of the Black Atlantic,” to chart the impact of black slavery on both sides of the Atlantic. Oberlin students complemented this event with, “Be Not Dismayed,” an exhibition of items from the university's archive of anti-slavery materials.

On October 20, 2019, we gathered with hundreds of congregants and friends at Riverside Church for the 400 Years of Inequality Project's Solemn Occasion, “Stolen Hands, Stolen Lands” (23). This three-and-a-half-hour long event included dancing, drumming, and song. Faith leaders offered libations, prayers and other ceremonies to honor the ancestors and affirm the commitment to equality at the moral and ethical heart of their traditions. An ensemble of performers read selections from the historical record: first person accounts of First Nation's leaders, activists and poets; appeals for freedom from enslaved and formerly enslaved people; and, articulations of justice from within justice struggles focused on the rights of women, people with disabilities, undocumented individuals, workers, and LGBTQ+ groups.

## Seven Sins

As we had envisioned, Rev. Dr. William Barber II delivered the homily, “Stolen Hands, Stolen Lands.” The sermon placed the United States on trial. Reverend Barber made the case that “seven sins” of the American colonies encoded structures of dehumanization that define the present. Movements for justice and liberation will need to dismantle these structures. “To name our sins,” he explained in his preamble, “is to tell the story of how we got here, but also it is to say we refuse to go along with sin even for a little while. Judgment is an opportunity to ask honestly whether we're going to repent and rebirth America again.”

These are the Seven Sins Rev. Barber urges us to reckon with if we are to take seriously the systemic racism we have inherited borne out of a system of slavery:

1. *Bad Biology* is the “naturalizing” and de-historicizing of inequality so that it might appear to be an inevitable outcome of the natural order. Barber cites a study by researchers at the Mailman School of Public Health at Columbia University who estimate that 250,000 people die each year from poverty (24). The construction of race in its weaponized form as racism undergirds this system.
2. Segregation, specifically the “separate but equal” racist doctrine codified in the US Supreme Court's ruling in Plessy v. Ferguson is *Sick Sociology* (25). It is the conviction that we cannot live as we wish with some people as our neighbors. That, Barber explains, is why we create ghettos or incarcerate people en masse.
3. *Political Pathology* works to establish and enforce inequality. It operates in Jim Crow laws and gerrymandered districts (26). It is the gutting of the Voting Rights Act and the disenfranchising of 6.1 million people—one in every thirteen black men aged 30–34—due to felony convictions (27).
4. *Corruptible Courts* helped create chattel slavery and continue to abet and enable practices of political pathology. Barber tells of the black servant John Punch, who in 1640, fled bondage. Two fellow servants, who happened to be white, escaped with him. The white men had their terms of indenture lengthened by several years, while John Punch was ordered to serve his master for the rest of his natural life. Thus, the courts inscribed racial inequality onto the lives of those who were equal in other ways, including their desire for liberty.
5. *Evil Economics* argues that profits justify both means and ends. Barber referenced specifically the failure to prosecute the greed and corruption that caused the financial crisis of 2007 and permitted thousands to fall into poverty and low wealth. We glimpse his fusion politics when he observes that while a far greater percentage of people of color live in poverty, in the raw numbers, more white women and white people are poor and low wealth than any other demographic. A unified, multi-racial constituency of poor and low wealth people could wield great power.
6. To explain *Militia Madness*, Barber recounted the contradictory history of Bacon's rebellion. In 1676 indentured white servants and enslaved, indentured, and free blacks united to attack local Native nations to take their land. They then overthrew the colonial government in Virginia, and for a time, ruled the colony together. “Black and white servants had long conjoined and conspired,” observes Barber, “but this was a new and dangerous level of cooperation that crossed the color line.” *Militia Madness* works to frustrate and violently suppress such alliances. The Second Amendment codified this practice to ensure that white men would be able to raise militias against slave rebellions.
7. Rev. Barber holds his Christian brethren complicit with his seventh sin, *Heretical Ontology*. This is the crafting of a theological justification for inequality evident in ideologies of race and Manifest Destiny. It presents the stealing of land and labor as “God's work.” Heretical Ontology is summed up by the declaration: “God meant it this way.” The ontological status of this assertion provides cover to the other six sins.

As we listened to William Barber make his case, we could not anticipate specifically where we would find ourselves in just a few months, in the grips of a pandemic and taking to the streets to demand justice. The framework he provided, however, enabled us to name the problems when they occurred.

As evidence of *bad biology*, we observed the litany of denials, distractions, falsehoods, and arrogant magical thinking that led the CDC in the 1st weeks of the pandemic to turn down the WHO's offer to provide test kits, only to find that its self-designed tests were faulty (28). It was also apparent in the callous and cavalier dismissal of the risks faced by workers in meatpacking plants (29). That these are mostly people of color and immigrants only increases their disposability.

With the endorsement of *corruptible courts*, the war on immigrants intensified as border restrictions increased and asylum claims were denied. The Department of Justice ordered immigration courts to continued to operate despite Coronavirus cases in the courthouse (30). Under the cover of crisis, conservatives deepened their efforts to appoint compliant ideologues, particularly to the Supreme Court of the United States.

The vulnerability of workers inherent to the *evil economics* of neoliberalism came to the fore almost immediately. The most undervalued and underpaid workers, most of them people of color, became classified as “essential.” As confirmed by epidemiologists, the wages for this reclassification were increased risk for infection, illness, and death (31). The pandemic thrust to the fore and amplified the economic vulnerabilities underlying homelessness, a growing precarious workforce, rental distortion created by real estate speculation, student loan debt, food insecurity, digital divides, and so on. Comparable distortions exist in other countries. In the UK, COVID-19 morbidity and mortality has followed the social gradient: infections and death clustered in the country's more deprived and predominanty racial minority areas. Building on earlier analyses of health inequities in the UK presented in the Black Report of 1980 and the Acheson Report of 1998, Marmot and Allen track what they term the “causes of causes” of health inequities, including the impact of neoliberal austerity, structural racism and other forms of inequality (32). Despite multiple and detailed findings regarding the social determinants of health, governments in the UK and elsewhere in the world have refused to enact adequate and timely reforms (33). Comparable trends and government failures are evident in Brazil, India, and multiple other countries (34, 35).

In their effort to blame the epidemic away, the U.S. president and his apologists set communities against each other by blaming China for the pandemic (36). This conjunction of *political pathology* and *sick sociology* surely made us sicker in body, in spirit, and in community, and, therefore, far more vulnerable than we would have been had we united. Blaming others, fueling hatred, and stoking division is the core practice of political pathology. These actions transformed a life saving practice, wearing protective masks, into an ideological melee that brought us to the brink of insurrection multiple times until, in early January 2021, that brink was crossed. Thus, we saw the clear link between *political pathology* and *militia madness*. The warning signs were there when gun merchants reported higher than usual sales as people purchased new and additional weapons (37).

As indicated above, according to Rev. Barber *heretical ontology* binds the other structures together into what we term, the “ecology of inequality.” To succumb to heretical ontology is the greatest defeat of all for it means that we both accept and collude in the production and enforcing of inequality. This heresy blinds us to our shared vulnerability and the awareness that by protecting others, especially the most vulnerable, we support our collective health. Withholding protective resources, vaccines and health care from homeless men and women or people who are incarcerated is a heretical betrayal of our shared life.

We outline this analysis in the spirit with which Rev. Barber presented his sermon on the seven charges against the US, particularly his conviction that in “learning from the sins of the past … we might embrace a better future.” To signal this turn toward the future, he reminds us of James Baldwin's charge: “We made the world we're living in and we have to make it over again” (38). In this period of crisis, we confront a most American of paradoxes: our deeply exercised capacity for, on the one hand, discrimination, exclusion, privilege, and dehumanization—we are not, as some in power claim, “all in this together”—and, on the other, the spirit of justice, equity, emancipation, and horizontalism—indeed we are “all in this together” if our intention is our collective well-being. Within this paradox lie crucial questions of spirit and being, questions of the meaning of existence, questions of community and society. We have an opportunity to look at who we are, to name the problem, and to envision who we might become.

In 1989, reflecting on the “black condition,” Cornel West suggested that, for black people, having long lived in crisis at, “the ragged edges of the Real, of Necessity, not being able to eat, not having shelter, not having health care,” had provided, “a strong sense of reality” (1). This oppressive condition in which life is bound by Necessity on one side and state and supremacist violence on the other is the reality, “that one cannot *not* know.” Rev. Barber is rooted in the black prophetic tradition that strives to name this reality, to make it evident. The uprisings following the killing of George Floyd were another prophetic edition. And it seems, “perhaps,” that others are waking up, that a critical mass is cognizant of the “ragged edges of the Real, of Necessity.” And, having woken up, are we possibly ready to take action. “I am committed to working with you all to build a moral fusion coalition in the 21st century,” announced Rev. Barber at the conclusion of his sermon. Then he asked: “Is there anybody else in here ready to build?”

## Data Availability Statement

The datasets generated for this study can be found in online repositories. The names of the repository/repositories and accession number(s) can be found at: www.400yearsofinequality.org.

## Author Contributions

All authors participated in the project described here, conceptualized the manuscript, and contributed to the writing.

## Conflict of Interest

The authors declare that the research was conducted in the absence of any commercial or financial relationships that could be construed as a potential conflict of interest.
